# The utility of biomarkers in diagnosis of aspirin exacerbated respiratory disease

**DOI:** 10.1186/s12931-018-0909-6

**Published:** 2018-10-30

**Authors:** Suzy A. A. Comhair, Grazyna Bochenek, Sara Baicker-McKee, Zeneng Wang, Tomasz Stachura, Marek Sanak, Jeffrey P. Hammel, Stanley L. Hazen, Serpil C. Erzurum, Ewa Nizankowska-Mogilnicka

**Affiliations:** 1 Cleveland Clinic, Lerner Research Institute, 9500 Euclid Avenue, NB2-40, Cleveland, OH 44195 USA; 2 Department of Internal Medicine, Faculty of Medicine, Jagiellonian University Medical College, Krakow, Poland; 3 Respiratory Institute, Cleveland Clinic, Cleveland, USA

**Keywords:** Asthma, BromoTyrosine, Leukotriene, AERD

## Abstract

**Background:**

Aspirin-exacerbated respiratory disease (AERD) is a distinct eosinophilic phenotype of severe asthma with accompanying chronic rhinosinusitis, nasal polyposis, and hypersensitivity to aspirin. Urinary 3-bromotyrosine (uBrTyr) is a noninvasive marker of eosinophil-catalyzed protein oxidation. The lack of in vitro diagnostic test makes the diagnosis of AERD difficult. We aimed to determine uBrTyr levels in patients with AERD (*n* = 240) and aspirin-tolerant asthma (ATA) (*n* = 226) and to assess whether its addition to urinary leukotriene E_4_ (uLTE_4_) levels and blood eosinophilia can improve the prediction of AERD diagnosis.

**Methods:**

Clinical data, spirometry and blood eosinophilis were evaluated. UBrTyr and uLTE_4_ levels were measured in urine by HPLC and ELISA, respectively.

**Results:**

Both groups of asthmatics (AERD, n = 240; ATA, n = 226) had significantly higher uBrTyr, uLTE_4_ levels, and blood eosinophils than healthy controls (HC) (*n* = 71) (*p* < 0.05). ULTE_4_ levels and blood eosinophils were significantly higher in AERD as compared to ATA (*p* = 0.004, *p* < 0.0001, respectively). whereas uBrTyr levels were not significantly different between both asthma phenotypes (*p =* 0.34). Asthmatics with high levels of uBrTyr (> 0.101 ng/mg Cr), uLTE_4_ levels (> 800 pg/mg Cr) and blood eosinophils (> 300 cells/ul) were 7 times more likely to have AERD.. However, uBrTyr did not increase the benefit for predicting AERD when uLTE_4_ and blood eosinophils were already taken into account (*p* = 0.57).

**Conclusion:**

UBrTyr levels are elevated both in AERD and ATA as compared to HC, but they could not differentiate between these asthma phenotypes suggesting a similar eosinophilic activation. The addition of uBrTyr to elevated uLTE4 levels and blood eosinophils did not statistically enhance the prediction of AERD diagnosis.

## Background

Around 7% of asthmatics are hypersensitive to aspirin and other non-steroidal anti-inflammatory drugs (NSAIDs) [1]. This asthma phenotype, referred to as Aspirin-Exacerbated Respiratory Disease (AERD), is characterized by the presence of usually severe asthma, chronic rhinosinusitis with nasal polyposis, and acute asthma attacks with naso-ocular symptoms after ingestion of aspirin and other NSAIDs [2]. Previous studies have shown that patients with AERD have increased emergency department visits, hospitalizations, and corticosteroid bursts compared to those with aspirin-tolerant asthma (ATA) [3, 4]. About half of AERD patients have severe asthma that requires chronic treatment with high dose of inhaled corticosteroids or oral corticosteroids to control the disease [4, 5].

AERD is an inflammatory condition of the upper and lower airway characterized by increased eosinophils and mast cells. Mechanistic studies have shown that AERD is linked to abnormalities/dysregulation of the cyclooxygenase (COX) and lipooxygenase (LOX) pathway [2, 6]. It has been suggested that abnormal regulation of 5-LOX and leukotriene C_4_ synthase (LTC4S) pathways that results in increased production of cysteinyl leukotrienes (CysLTs) leads to asthmatic attacks [2, 7, 8]. Urinary leukotriene E_4_ (uLTE_4_), a stable CysLTs metabolite, is used to measure their systemic production [9] and can be helpful in differentiating AERD from ATA [10, 11]. Sources of LTE_4_ include many cells of the upper and lower airways such as eosinophils, mast cells, basophils, as well as macrophages, platelets, and neutrophils [12].

Eosinophils are important effector cells in asthma and even more so in AERD. Blood eosinophilia is well-recognized biomarker of active inflammation in asthma [13, 14]. Activated eosinophils degranulate to release among others eosinophil peroxidase (EPO), which is unique in its ability to convert respiratory burst-generated hydrogen peroxide into hypobromous acid, a reactive brominating oxidant that modifies protein tyrosine residues, forming 3-bromotyrosine (BrTyr) [15, 16]. BrTyr, a biochemical fingerprint of eosinophil activation as a highly stable product can be detected in urine.

Currently, oral aspirin challenge is the gold standard to confirm the diagnosis of AERD [17, 18]. However, this test is time-consuming, can cause adverse reactions, and is unsuitable in severe asthma patients [17]. Therefore, noninvasive methods that could assist in diagnosis are urgently needed.

To our knowledge, there is no generally recognized, clinically available in vitro test or biomarker to determine the presence of AERD.

The purpose of this study was to determine uBrTyr levels in patients with AERD and ATA and to explore if uBrTyr might predict AERD. Moreover, we wanted to test whether adding elevated uBrTyr levels to elevated uLTE_4_ levels and blood eosinophil counts could improve the prediction of AERD.

## Methods

### Study population

Patients with asthma were recruited from the outpatient clinic of the Department of Internal Medicine, Jagiellonian University Medical College, Krakow. All patients were clinically stable without any asthma exacerbations within the 6 weeks preceding the study. All data and sample collection have been previously described [11].

The diagnosis of AERD was determined through typical clinical presentation and at least 1 asthma attack after ingestion of aspirin or another NSAID in the past. This diagnosis was confirmed by a positive aspirin challenge in the majority of patients. However, clinical safety limitation prohibited a small group of AERD patients (16.7%, *n* = 40)) with very severe asthma to undergo aspirin challenge. Their aspirin hypersensitivity was confirmed based on their medical history with previous reactions related to ingestion of NSAIDs well documented in medical records. Removal of these severe asthmatics could introduce additional bias in characteristics of AERD phenotype, for which severe steroid dependent asthma is very typical.

All ATA patients had used aspirin without any adverse effects. Healthy control subjects (HC) without history of asthma, allergy, and any hypersensitivity reactions to NSAIDs were enrolled for comparison of baseline values of inflammatory biomarkers.

Written informed consent was obtained from all participants under a protocol approved by the Jagiellonian University Ethics Review Committee.

Evaluation of asthma was determined by the National Asthma Education and Prevention Program (NAEPP) EPR-3 guidelines [19].

Asthma control was assessed by the Asthma Control Test (ACT) [20].

### Study procedures

Spirometry (MasterScreen, Jaeger, Wurzburg, Germany) was carried out at baseline and after administration of 4 puffs of salbutamol. The best of 3 repeatable forced expiratory maneuvers was recorded. The percent predicted values of FEV_1_ and FEV_1_/FVC were automatically calculated.

Blood eosinophil counts were calculated using a Fuchs-Rosenthal chamber.

### Urine analysis

Morning urine samples were collected after a 2 h accumulation of urine in the bladder which allows for less variation in urine creatinine levels [10].

Urine creatinine was measured by enzymatic method using automated chemical analyzer COBAS Integra 400 plus (Roche Diagnostics USA, Indianapolis, IN).

Urinary BrTyr was assayed using stable isotope dilution High Performance Liquid Chromatography (HPLC) with on-line electrospray ionization tandem mass spectrometry. First, a solid phase extraction was performed. Briefly 20 μl internal standard composed of 0.5 μM synthetic [^13^C_6_]-3-bromotyrosine, 1 mM [^13^C_9_, ^15^N] tyrosine, and 1 mM creatinine-d_3_ (Cambridge Isotope Laboratories) was spiked into 200 μl urine followed by acidification with 1 ml 0.1% formic acid. The acidified urine was loaded to a Biotage® auto solid phase extraction system with 3 ml DSC- 18 column used. DSC-18 column was balanced with 2 × 3 ml methanol and then 2 × 3 ml 0.1% formic acid, washed with 2 × 3 ml 0.1% formic acid, and the product was eluated with 3 ml 0.1% formic acid in 30% methanol. The product was dried under SpeedVacuum, and then resuspended in 100 μl H_2_O. Secondly, LC/MS/MS was used to quantify urinary BrTyr. Five microliters of the extraction was analyzed by injection onto a Titan™ C18 UHPLC Column (1.9 μm particle size, L × I.D. 10 cm × 2.1 mm, Supelco) at a flow rate of 0.4 ml min^− 1^ using a 2 Shimadzu LC-20 AD Nexera CL pump system, SIL-30 AC MP CL autosampler interfaced with an Shimadzu 8050 mass spectrometer. A discontinuous gradient was generated to resolve the analytes by mixing solvent A (0.2% formic acid in water) with solvent B (0.2% formic acid in methanol) at different ratios starting from 0% B for 3 min, then linearly to 100% B over 3.5 min, then hold for 3 min, and then back to 0% B. [^13^C_9_, ^15^N_1_]-tyrosine was included to simultaneously monitor for potential artificial generation of analyte. 3-bromotyrosine, creatinine, and their respective internal standards [^13^C_6_]-3-bromotyrosine and creatinine-d_3_ were monitored using electrospray ionization in positive-ion mode with multiple reaction monitoring (MRM) of precursor and characteristic product-ion transitions of *m/z* 260 → 135, 114 → 44, 266 → 141 and 117 → 47 amu, respectively. The parameters for the ion monitoring were optimized automatically. Nitrogen (99.95% purity) was used as the source, and helium was used as collision gas. Various concentrations of nonisotopically-labeled 3-bromotyrosine were spiked into control urine to prepare the calibration curves for quantification of 3-bromotyrosine. The internal standard [^13^C_6_]-3-bromotyrosine was used for quantification as well as to calculate recovery rate of TMAO (which was > 80% based on separate control studies). Under the conditions employed for the assay, no artificial bromination was detected. Results of urinary BrTyr were expressed in nanograms per mg of creatinine.

ULTE_4_ was measured in unpurified urine samples by direct enzyme immunoassay (Cayman Chemical, Ann Arbor, MI). Supernatants of freshly collected spot urine samples were stored at − 80°Celsius for not longer than 6 months. After thawing at 4 °C, urine was diluted in phosphate buffered saline (1:10). The measurement was repeated using 1:30 dilution of urine for samples in which the LTE_4_ concentration exceeded the uppermost calibrator concentration of the assay (1000 pg/mL). Results of urinary LTE_4_ measurements were expressed in picograms per mg of creatinine.

### Statistical analyses

Quantitative characteristics of the study population were described using mean and standard error, while categorical characteristics were described using means and percents. Groups were compared with respect to baseline quantitative characteristics and marker levels among the groups with t-tests and analyses of variance for approximately normally distributed variables, while the Wilcoxon rank-sum test was used with respect to variables demonstrating non-normal distributions. Groups were compared using the likelihood-ratio χ^2^ test with respect to categorical variables. To evaluate uBrTyr, uLTE_4_, and blood eosinophils as biomarkers for AERD diagnosis, cut-points were used. A receiver operating characteristic (ROC) analysis was used to identify an optimal cut-point for uBrTyr and uLTE_4_. A cut-point for blood eosinophils > 300 cells/μL was based on published data [21, 22]. Logistic regression was used to estimate odds ratios for the identification of AERD (vs ATA) with respect to the dichotomized forms of the biomarkers. The logistic regression analyses were also performed with covariate adjustment for baseline FEV_1_%, chronic rhinosinusitis, and any steroid use was performed with the logistic regression modeling. The incremental predictive ability of each marker in the regression model was assessed using areas under the ROC curves estimated with 10-fold cross-validation for the full model and for models excluding markers individually. Correlations among quantitative variables were assessed using Spearman’s rank-sum correlation coefficients, which are suitable for data with any distribution. A *p*-value less than or equal to 0.05 was defined as statistically significant.

## Results

### Subject characteristics

The study included 240 patients with AERD, 226 patients with ATA, and 71 HC. Almost the same population has been previously described [11]. The clinical characteristics of the study subjects are shown in Table 1. Age and gender were distributed significantly differently in asthmatics, i.e., AERD and ATA when compared to (HC), but no difference between AERD and ATA was found. (Table 1). The AERD subjects had worse lung functions and significantly longer asthma duration than the ATA subjects (Table 1). Medication use was different between the AERD and the ATA groups. No difference was found with respect to the ACT scores (Table 1).

**Table 1:** Participant characteristics

	AERD [*n* = 240]	ATA [*n* = 226]	Control [*n* = 71]	*p*-value (ANOVA)	T-Test
Demographics					
Age (yrs)	49.3 [0.8]	49.7 [1.0]	44.3 [1.6]	0.008	
Gender [M/F]	74/166	68/158	35/36	0.007	
BMI (kg/m^2^)	26.7 [0.3]	27.1 [0.4]	25.6 [1.0]		
Duration of asthma (yrs)	18.8 [0.8]	15.2 [1.0]	n/a		< 0.0001
ACT; uncontrolled, n [%]	108 [46]	98 [46]	n/a		
Medication use					
High dose ICS/Oral CS, n (%)	63 (26)	37 (18)	n/a		0.03
High dose ICS > 500 (μg/d), n (%)	62 (26)	52 (25.5)	n/a		
Low dose ICS ≤500 (μg/d), n (%)	78 (33)	92 (44.7)	n/a		0.008
No ICS or Oral CS, n (%)	3 (14)	25 (12.4)	n/a		
Lung functions					
FEV_1_% predicted	79.8 [1.3]	86.6 [1.4]	n/a		0.0003
FEV_1_ %FVC	68.8 [0.7]	74.8 [0.7]	n/a		< 0.0001

Mean (SEM); Definition of abbreviations: *AERD* Aspirin-Exacerbated Respiratory Disease, *ATA* Aspirin Tolerant Asthma, *M* Male, *F* Female, *BMI* Body mass index, *FEV*_*1*_ Forced expiratory volume in 1 s, *FVC* Forced vital capacity, *CS* Corticosteroidsm, *ICS* Inhaled Corticosteroids

### Asthma biomarkers

**Fig. 1:**
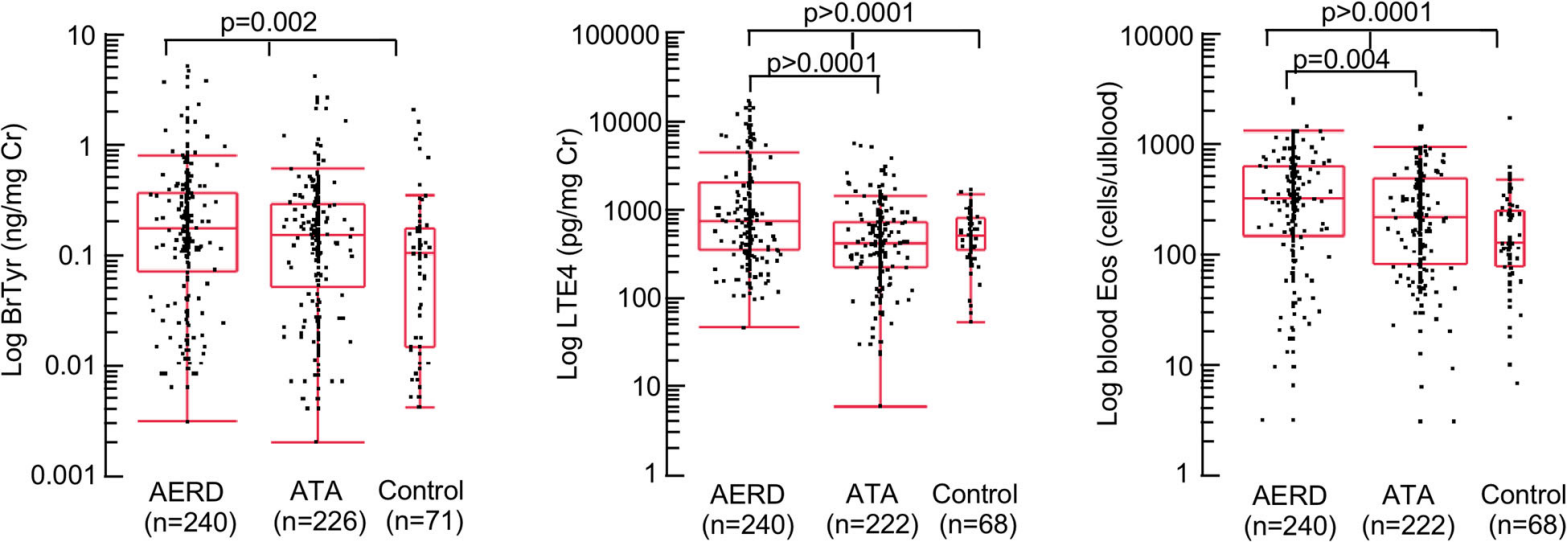
Asthma biomarkers in AERD, ATA and control subjects. Evaluation of urinary BrTyr, urinary LTE_4_, and blood eosinophils in patients with Aspirin-Exacerbated Respiratory Disease (AERD) (*n* = 240), patients with Aspirin-Tolerant Asthma (ATA) (*n* = 222), and control subjects (*n* = 68). All markers were significantly different between the three groups (ANOVA, *p* < 0.05). Urinary BrTyr was the only biomarker not significantly different between ATA and AERD (*p* > 0.05)

Both AERD and ATA groups had significantly higher levels of uBrTyr, uLTE_4_, and blood eosinophils than HC (Fig. 1). Furthermore, uLTE_4_ levels and blood eosinophils were significantly higher in AERD as compared to ATA (*p* < 0.0001, *p* = 0.004, respectively), whereas uBrTyr levels were not significantly different between these two asthmatic phenotypes (*p =* 0.3406) (Fig. 1).

UBrTyr and uLTE_4_ levels correlated with each other both in the AERD [*R* = 0.160, *p* = 0.01] and the ATA [*R* = 0.151, *p* = 0.02] groups. Blood eosinophils correlated significantly with uLTE_4_ levels in both asthma phenotypes [ATA: *R* = 0.165, p = 0.01; AERD: *R* = 0.276, p < 0.0001], whereas no such correlation was found with eosinophil counts and uBrTyr levels.

Both uBrTyr and uLTE_4_ levels did not correlate with severity of airway obstruction measured by FEV_1_% predicted and FEV_1_%FVC in either asthma phenotype, while only a borderline correlation was found between FEV_1_%FVC and uLTE_4_ in ATA [*R* = 0.140, *p* = 0.05].

**Fig. 2:**
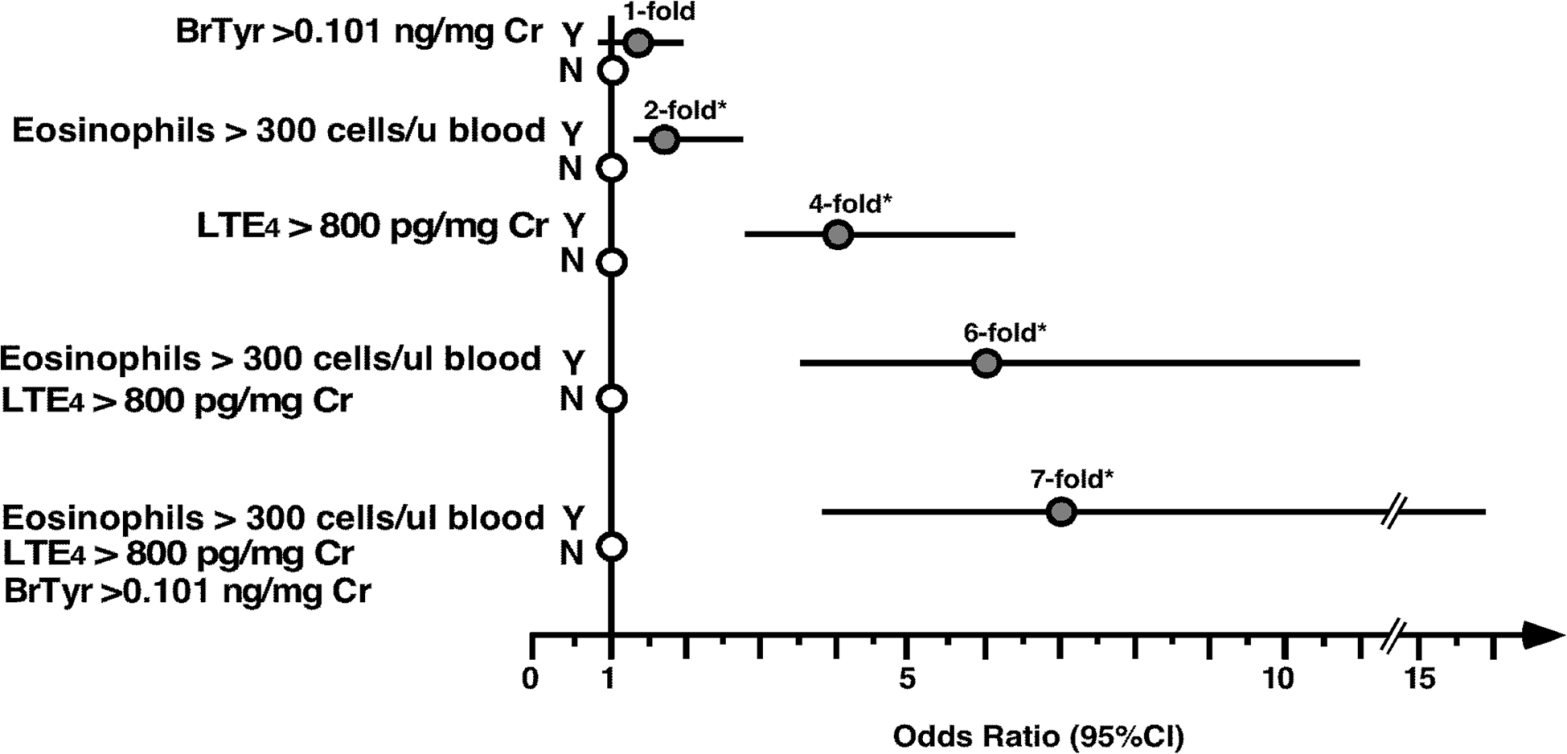
Odds ratios and 95% confidence intervals for the association between the presence of elevated marker levels, individually and in combination, versus the diagnosis of AERD. Results shown represent the ORs *(filled circles)* and 95% CI *(lines)* of having AERD versus ATA *(open circles)* for participants with high levels of urinary BrTyr and/or high levels of blood eosinophils, and/or high levels of urinary LTE4 compared with participants with low levels of these markers. *Asterisk* indicates *P* < 0.05

To evaluate if studied biomarkers have the ability to predict an AERD diagnosis, values for each biomarker were dichotomized at cutoffs derived from ROC curve analyses. The cutoff value for uBrTyr was 0.101 ng/mg Cr (specificity 37%, sensitivity 70%), and the cutoff value for uLTE_4_ was 800 pg/mg Cr (specificity 81%, sensitivity 50%). A high level of uBrTyr did not confer greater odds of having AERD (OR 1.3; 95% Cl 0.9–2.0, *p* = 0.15) (Fig. 2). The odds ratio for predicting AERD with respect to blood eosinophils > 300 cells/uL was 1.8 (95% CI 1.2–2.6, *p* = 0.003) (Fig. 2). A high level of uLTE_4_ > 800 pg/mg Cr conferred 4.1-fold odds of having AERD (95% CI 2.7–6.3; *p* < 0.0001). The combination of high blood eosinophils together with high uLTE_4_ levels increased the odds for AERD diagnosis 6.0-fold (95% CI 3.4–10.9, p < 0.0001), with frequency of AERD of 79.6% (74/93) among asthmatics with both characteristics versus 39.5% (77/195) among patients with neither characteristic. Asthmatics with a combination of high blood eosinophil counts, uLTE_4_ levels, and uBrTyr levels had 7.1 times greater odds to have AERD than patients with none of the biomarkers elevated (95% CI 3.4–15.8, p < 0.0001) (Fig. 2), with frequency of AERD of 83.1% (59/71) among asthmatics with all three characteristics versus 41.0% (32/78) among patients with none of the characteristics. In the multivariable model with all 3 biomarkers, uBrTyr did not show statistically significant evidence of an additive benefit for predicting AERD when uLTE_4_ and blood eosinophils were already taken into account [adjusted odds ratio 1.1, 95% CI 0.8–1.7, *p* = 0.57].

When performing further covariate adjustment for baseline FEV_1_%, chronic rhinosinusitis, and steroid use, the combination of high blood eosinophils together with high uLTE_4_ levels increased the odds for AERD diagnosis 3.5-fold (95% CI 1.7–7.4, *p* = 0.0007), which was smaller than the 6.0-fold estimate without the additional covariate adjustments. Asthmatics with a combination of high blood eosinophil counts, uLTE_4_ levels, and uBrTyr levels had covariate-adjusted 7.7 times greater odds to have AERD than patients with none of the biomarkers elevated (95% CI 2.8–24.7, *p* = 0.0002), which was slightly higher than the 7.1-fold estimate obtained without the additional covariate adjustments. The additive benefit of uBrTyr for predicting AERD when uLTE_4_ and blood eosinophils were already taken into account also jumped upward a bit [adjusted odds ratio 1.5, 95% CI 0.9–2.5, *p* = 0.09] when adjusting for the additional covariates, compared to the 1.1-fold increase estimated without the additional adjustments. The adjustment for steroid use was also performed with respect to only high dose steroid use, but the odds ratio estimates for the biomarkers was virtually identical to those obtained with the adjustment for any steroid use. Using ROC curves, 10-fold cross-validated AUC estimates were 0.82 for the full model and for models excluding either uBrTyr or blood eosinophils, while the exclusion of uLTE_4_ yielded an estimate of 0.79. This is not surprising given that among the biomarkers, only uLTE_4_ levels yielded a statistically significant association with AERD when adjusting for the other biomarkers and the selected covariates.

The cutpoint for uBrTyr level to predict asthma regardless of phenotype was > 0.17 ng/mg Cr (specificity, 79%; sensitivity, 45%). Subjects with baseline uBrTyr levels > 0.17 ng/mg Cr were 3.1 times (95% CI 1.7–5.8], *p* > 0.0002) as likely to have asthma.

## Discussion

Aspirin challenge is currently considered as the gold standard and the only reliable method for the diagnosis of AERD. However, due to certain limitations of this procedure, an intensive search for in vitro diagnostic biomarkers useful in identifying patients with AERD is underway.

Novel biomarkers such as serum periostin [23], plasma eosinophil-derived neurotoxin [24], serum levels of LTE_4_, and LTE_4_/PGF_2_ alfa ratio [25] have been suggested but are not routinely done as in vitro diagnostic tests. They require verification in further studies.

As the imbalance of arachidonic acid metabolism and increased inflammation are important pathophysiologic features of AERD, we investigated if a panel of biomarkers reflecting these imbalances could be used for the prediction of this asthma phenotype. The current study confirmed previous reports that uLTE_4_ levels and blood eosinophil counts are increased in AERD patients as compared with ATA patients [9, 11, 26–28]. Additionally, it revealed that both elevated blood eosinophil count and increased uLTE_4_ levels separately enhanced the chance of AERD diagnosis 1.8-fold and 4.2-fold, respectively. The novel finding is that the combination of these two biomarkers powers that prediction 6.0-fold.

Recently, Bochenek et al. demonstrated that a set of clinical parameters comprising nasal polyps, upper airway symptoms, nasal corticosteroid treatment, asthma exacerbations, FEV_1_% predicted, and age of asthma onset had a superior accuracy in the prediction of AERD diagnosis to the measurement of uLTE_4_ level alone [11]. Addition of high uLTE_4_ level to clinical parameters slightly enhanced the prediction of such diagnosis.

Ban et al. analyzed a metabolite profile that involved the arachidonic acid pathway for discriminating AERD from ATA and revealed that serum levels of LTE_4_ and even more so LTE_4_/PGF_2_α ratio can be potential in vitro diagnostic biomarkers for AERD [25]. Thus, similarly to the current study, the combination of two biomarkers together improved the diagnostic value of the test.

Eosinophils and mast cells play an important role in the pathogenesis of AERD [7]. Studies have suggested that eosinophils in AERD are driving cysteinyl leukotrienes overproduction and eosinophils proteins, e.g. ECP increased following aspirin provocation fast [29, 30]. Previous findings showed that uBrTyr can be used as (1) a molecular fingerprint of eosinophil activation; (2) a predictor of asthma and asthma exacerbation; and (3) a biomarker for asthma severity and corticosteroid responsiveness [15, 16, 31–34]. Despite serving as a marker for eosinophil activation, uBrTyr levels did not correlate with the amount of blood eosinophils found in either the AERD or in the ATA group. The study by Mita et al. on very small groups of patients with AERD (*n* = 12), ATA (*n* = 12), and control subjects (*n* = 18) showed also that higher uBrTyr levels in asthmatics were not related to the presence of aspirin sensitivity, but rather to their asthma alone [35]. As shown previously with asthmatic adult and pediatric populations [31, 32, 35], in our cohort of adult asthmatics uBrTyr levels were also increased in asthma when compared with HC and could be used as a biomarker of asthma. However, a higher uBrTyr level alone cannot distinguish patients with AERD from patients with ATA, and thus it cannot be used as a single parameter helpful in the diagnosis of AERD. The multivariable modeling conducted presently demonstrates a slight predictive benefit of uBrTyr level in addition to the use of elevated blood eosiniphils and elevated uLTE_4_ that is enhanced with covariate adjustment for FEV_1_%, chronic rhinosinusitis, and steroid use, though not reaching a magnitude of statistical significance (*p* = 0.09).

## Conclusion

This study reveals that uBrTyr levels are increased both in AERD and in ATA but cannot identify AERD. This suggested that the activation and the role of eosinophils in the controlled asthma is similar regardless aspirin hypersensitivity. The addition of uBrTyr to elevated uLTE4 levels and blood eosinophils did not statistically enhance the prediction of AERD diagnosis.14.

## Abbreviations

AERD: Aspirin-exacerbated respiratory disease
ATA: Aspirin-tolerant asthma
COX: Cyclooxygenase
CysLTs: Cysteinyl leukotrienes
HC: Healthy controls
HPLC: High Performance Liquid Chromatography
LOX: Lipoxygenase
LTC4S: Leukotriene C_4_ synthase
UBrTyr: Urinary 3-bromotyrosine
ULTE_4_: Urinary leukotriene_4_ E
